# Offsetting unabated agricultural emissions with CO_2_ removal to achieve ambitious climate targets

**DOI:** 10.1371/journal.pone.0247887

**Published:** 2021-03-17

**Authors:** Nicoletta Brazzola, Jan Wohland, Anthony Patt

**Affiliations:** Department of Environmental Systems Science, Institute for Environmental Decisions, ETH Zurich (Swiss Federal Institute of Technology), Zurich, Switzerland; Texas A&M University, UNITED STATES

## Abstract

The Representative Concentration Pathway 2.6 (RCP2.6), which is broadly compatible with the Paris Agreement’s temperature goal by 1.5–2°C, contains substantial reductions in agricultural non-CO_2_ emissions besides the deployment of Carbon Dioxide Removal (CDR). Failing to mitigate agricultural methane and nitrous oxide emissions could contribute to an overshoot of the RCP2.6 warming by about 0.4°C. We explore using additional CDR to offset alternative agricultural non-CO_2_ emission pathways in which emissions either remain constant or rise. We assess the effects on the climate of calculating CDR rates to offset agricultural emission under two different approaches: relying on the 100-year global warming potential conversion metric (GWP100) and maintaining effective radiative forcing levels at exactly those of RCP2.6. Using a reduced-complexity climate model, we find that the conversion metric leads to a systematic underestimation of needed CDR, reaching only around 50% of the temperature mitigation needed to remain on the RCP2.6 track. This is mostly because the metric underestimates, in the near term, forcing from short-lived climate pollutants such as methane. We test whether alternative conversion metrics, the GWP20 and GWP*, are more suitable for offsetting purposes, and found that they both lead to an overestimation of the CDR requirements. Under alternative agricultural emissions pathways, holding to RCP2.6 total radiative forcing requires up to twice the amount of CDR that is already included in the RCP2.6. We examine the costs of this additional CDR, and the effects of internalizing these in several agricultural commodities. Assuming an average CDR cost by $150/tCO_2_, we find increases in prices of up to 41% for beef, 14% for rice, and 40% for milk in the United States relative to current retail prices. These figures are significantly higher (for beef and rice) under a global scenario, potentially threatening food security and welfare. Although the policy delivers a mechanism to finance the early deployment of CDR, using CDR to offset remaining high emissions may well hit other non-financial constraints and can thus only support, and not substitute, emission reductions.

## Introduction

To limit global warming well below 2°C, mitigation of CO_2_ emissions alone will not be sufficient [1]. Currently roughly one-third of climate forcing is caused by non-CO_2_ climate pollutants [2] and agriculture causes a large fraction of these emissions [3]. Taken altogether, food systems contribute somewhere between 21–37% of total anthropogenic greenhouse gas emissions [4, 5]. Moreover, the relative importance of non-CO_2_ agricultural emissions will likely increase as CO_2_ emissions decline to net zero [1, 6–8]. Methane emissions, contributing around a quarter of the 2011 total anthropogenic radiative forcing (cf. Figure 8.15 IPCC AR5 WGI [3]), are largely from anthropogenic sources, with half of them from agriculture (e.g. due to livestock production and rice cultivation) [9–12]. Nitrous oxide, contributing to around 6% of the total greenhouse gases radiative forcing [3], also results primarily from agricultural activities [3], such as the use of inorganic fertilizer and the cultivation of nitrogen-fixing crops [13].

In the absence of transformative policies and technical mitigation options [11], agricultural non-CO_2_ emissions would reduce the remaining carbon budget so as to result in infeasible emission reduction targets for CO_2_ [1, 6–8, 14, 15]. Although agricultural productivity shows signs of decoupling from its emissions [16], an ever-growing demand for food production has caused an increase in the absolute agricultural-sector emissions over recent decades [17]. Under business-as-usual scenarios, this growth will continue due to dietary changes linked with increased income per capita, and by 2050 agricultural emissions would be 30–40% higher than today [4, 6, 11, 13, 18]. By contrast, all IPCC scenarios for the agricultural sector that limit warming below 2°C contain non-CO_2_ emissions declining by 11–30% relative to 2010 already by 2030 [1, 19]. The IPCC scenarios achieve the needed agricultural non-CO_2_ emission reductions (1–2 GtCO_2_-equivalent per year (GtCO_2_-eq/yr) already by 2030 [1, 11, 19]) through improved agricultural management practices and dietary shifts away from emissions-intensive livestock products [1]. These rates of emission reductions are within the estimates of technically feasible supply-side (1.6–4.6 GtCO_2_-eq/yr by 2030 [3]) and demand-side (1–8 GtCO_2_-eq by 2050 [20–22]) mitigation potentials. Additional mitigation could result from novel technologies (e.g. novel plant-based and synthetic proteins, methane inhibitors and vaccines in livestock, and nitrification inhibitors) [1, 4], although these are sufficiently immature to be excluded from the IPCC scenarios.

Albeit technically feasible, curbing non-CO_2_ agricultural emissions faces several challenges. Plausible agricultural development pathways with mitigation co-benefits can contribute only around 0.2–0.4 GtCO_2_-eq of avoided emissions by 2030, just 10–20% of what needed to limit warming well below 2°C [11]. For the livestock sector, it is estimated that only around 10% of the technically possible mitigation might be economically viable at abatement costs of $50/tCO_2_eq [22]. Large up-front investments would be needed to enable technological supply-side mitigation [23], should it prove difficult to fully exploit the large potential of demand-side mitigation, such as dietary shifts. Although reductions in meat consumption alone could significantly decrease agricultural emissions, the current trend is the opposite of this [24]. Due to the cultural, affective, and behavioural value of food, people’s willingness to change meat consumption behaviour is low [25–27], and policies would be needed to incentivize dietary shifts.

Policies to reduce the emission footprint of diets could include the pricing of emissions. Since many food products have a low price elasticity of demand and limited options to reduce emissions through low-cost changes in farm systems, large increases in prices would be necessary to achieve modest decreases in emissions [22, 28], exposing this kind of policies to socio-political resistance [24, 29]. Furthermore, applying a globally uniform price to agricultural emissions could lead to trade-offs between ambitious climate targets and food security [30], since stringent climate policies increase food prices in the short run. Achieving the 1.5°C target without introducing food security support policies could lead to a rise in undernourishment of 80–300 million people in 2050 [30–32].

Given these challenges to achieve rapid reductions in agricultural emissions, a substantial gap between modelled emission pathways and reality appear imminent [15]. If agricultural emissions are not mitigated beyond current levels, the remaining carbon budget to reach the 1.5°C target is cut by more than half [33–35]. As a result, offsetting unabated agricultural emissions could become necessary to achieve climate targets in line with the Paris Agreement. Since technologies to sequester non-CO_2_ species from the atmosphere exist only at the conceptual level and the feasibility of their implementation is debated [1], offsetting agricultural emissions through additional CO_2_ negative emissions could become necessary.

Carbon Dioxide Removal (CDR) plays an important role in emissions pathways that are consistent with ambitious long-term temperature targets (below 2°C) [1, 3]. CDR encompasses different techniques with a varying degree of maturity, scalability, and removal potential [36]. Although heavily deployed in emissions scenarios compatible with the Paris Agreement’s temperature targets [1], knowledge of how to scale-up and internationally govern CDR as well as on its implications on the planet and society is lacking [36–42]. Existing carbon emission trading mechanisms have been proposed as main avenues to finance CDR [43–45], yet it is unclear how this would work to guarantee the offset of non-CO_2_ emissions. Polluters would have, in fact, an incentive to completely eliminating gross CO_2_ emissions if this results to be more cost-effective rather than paying a price on carbon reflecting the costs of CDR [46].

Offsetting non-CO_2_ greenhouse gas emissions via removal of CO_2_ requires defining a conversion metric between different emission species. Parties to the UNFCCC and to the Paris Agreement currently report aggregate CO_2_-equivalent emissions and removals of greenhouse gases using the 100-year time-horizon Global Warming Potential (GWP100) as a conversion metric [47]. GWP100 is a simple heuristic that accounts for the integrated change in radiative forcing–the perturbation of the Earth’s atmospheric energy balance, which leads to warming–over the 100 years following a pulse emission of a given climate pollutant, relative to the same quantity of CO_2_. In contrast to explicit radiative transfer modelling, GWP100 does not account for interactions between different species. A large body of literature highlighted shortcomings of assessing the warming effect of short-lived climate pollutants, such as methane, with GWP100 [48–53]. Yet, this inaccurate use of the metric remains widespread and with serious implications: the implementation of the Paris Agreement without adjustments to its emission metrics results in an uncertainty in temperature outcomes as high as 0.17°C [52]. Alternatives to GWP100 exist, spanning from Global Temperature Potential (GTP) metric (similarly comparing emissions pulses, but based on the ratio of warming amounts at the end of the time horizon [54]) to an alternative usage of the GWP metric (denoted GWP*) relating cumulative CO_2_ emissions to changes in the rate of emissions of short-lived climate pollutants [35, 55]. Huntingford et al. (2015) [56] showed that using GWP100 and GTP100 to substitute methane emissions with CO_2_ reductions, in the RCP2.6 scenario, leads to higher global mean temperatures than an equivalent methane emissions reduction. Lauder et al. (2013) [57] calculated that an one-off sequestration of one tonne of carbon offsets ongoing emissions by 0.9–1.05 kg methane per year.

In this study, we explore the use of CDR to offset agricultural emissions of methane and nitrous oxide overshooting the RCP2.6 budget. Although previous studies address the substitutability between more or less ambitious reductions of (agricultural) methane emissions and allowable CO_2_ emissions in general [15, 56], these substitutions have never been explicitly investigated in the context of offsetting via CDR. Reliable estimates of CDR requirements are essential to correctly offset the additional agricultural methane and nitrous oxide emissions. First, we test whether using the GWP100 yields the same CDR rates as explicit modelling, the latter based on the concept of effective radiative forcing (ERF). Second, we compare the use of two alternative metrics (GWP20 and GWP*) to offset methane. Finally, we explore the internalisation of the framework’s costs, such as with a tax on methane and nitrous oxide emissions. Unlike previous studies [22, 23, 28, 58], we do not focus on using the tax to reduce emissions through highly inelastic demand-side effects and changes in production systems, but rather on using the tax as a revenue-raising tool to fund CDR offsetting. As part of this, we assess the change in price, over current retail price benchmark for a United States and global scenario, of three exemplary agricultural products (beef, milk, and rice) in the presence of such offsetting.

## Methods

The RCP2.6 leads to an expected temperature increase by 2100 in the range of 1.5–2°C [59] and is hence an emission pathway broadly compatible with the Paris Agreement’s target. We investigate the amount of CDR required to achieve radiative forcing and consequently warming rates as in the RCP2.6 for alternative scenarios of agricultural emissions. Our reference scenario, the “SSP1-2.6”, achieves radiative forcing as in the RCP2.6 assuming agricultural emissions and CDR rates as in the SSP1-2.6 (“Sustainability–Taking the Green Road” reaching radiative forcing by 2.6Wm^-2^) [7, 60, 61]. In this scenario, methane emissions from the agricultural sector decrease by 56% by 2100 from their observed values in 2015, while agricultural nitrous oxide increases by 3.5%. The reduction in methane is slightly higher than the average reduction in agricultural and land use methane emissions of the IPCC scenarios compatible with the 1.5°C goal and limited overshoot (-48±20%). On the opposite, the 1.5°C scenarios contain on average larger reductions in nitrous oxide than the SSP1-2.6 (-23±40%) [19]. We additionally explored two alternative pathways of agricultural non-CO_2_ emissions: a “constant” scenario, where agricultural methane and nitrous oxide emissions remain stable at their 2015 levels, and a “worst-case” scenario, where they increase as in the baseline scenario of the Shared Socioeconomic Pathway with the least mitigation in the agricultural sector (the SSP3-7.0) [7]. We chose the SSP3-7.0 scenario to explore the upper boundary of offsetting requirements within the agricultural sector, since it is the SSP scenario with the highest challenges in both mitigation and adaptation in the land use sector [7]. The details of the different scenario constructions are summarized in Table 1.

**Table 1:** Details of alternative scenarios used in this study.

	“SSP1-2.6”	“Constant”	“Worst-case”
Agricultural methane	SSP1-2.6	Agricultural emissions constant at 2015 observations as long as they are higher than in SSP1-26 scenario	Agricultural emissions as in SSP3-7.0
Agricultural nitrous oxide	SSP1-2.6	Agricultural emissions constant at 2015 observations as long as they are higher than in SSP1-26 scenario	Agricultural emissions as in SSP3-7.0
Other species (including non- agricultural methane and nitrous oxide)	RCP2.6	RCP2.6	RCP2.6

**Fig 1:**
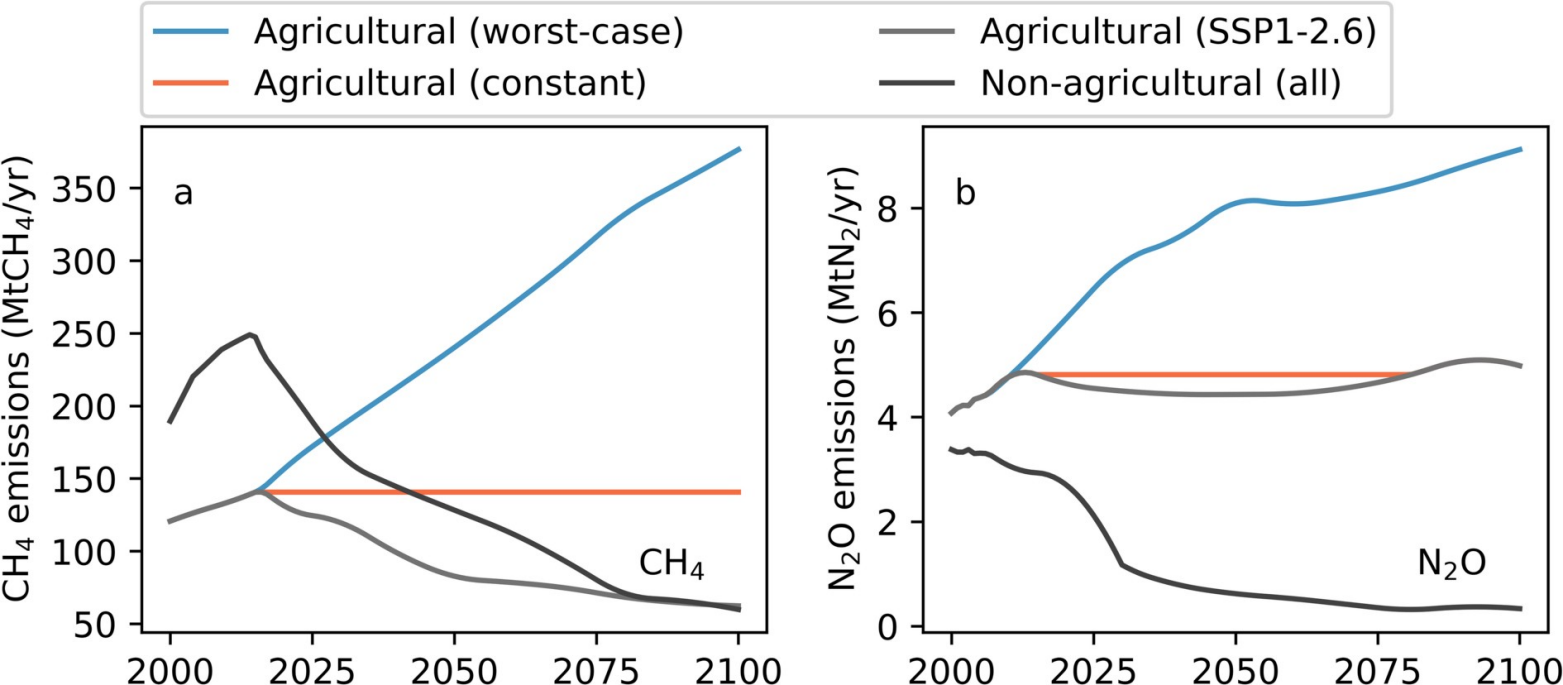
Methane (panel a, “CH_4_”) and nitrous oxide (panel b, “N_2_O”) emission pathways under the SSP1-2.6 and alternative scenarios (constant and worst-case emissions). Agricultural emissions differ between the three scenarios (SSP1-2.6, constant, and worst-case) while non-agricultural emissions are the same in all three scenarios (and corresponding to SSP1-2.6 emissions). The data used to reproduce this figure are publicly available in the IIASA SSP database [60–63].

Fig 1 depicts the methane and nitrous oxide emissions pathways associated with these scenarios. We use agricultural methane and nitrous oxide, as well as CDR rates, from the harmonized CMIP6 emissions data set [62, 63] from year 1765 to 2100 for the SSP1-2.6 (for the “SSP1-2.6” scenario) and from the SSP3-7.0 (for the “worst-case” scenario), as detailed in Table 1. We hold net emissions of climate-forcing species other than methane, nitrous oxide, and CO_2_, which is also affected by CDR, at levels envisioned by the RCP2.6 scenario.

To calculate the required CDR rates, we use two different approaches. The first approach (“GWP100”), depicted in Eq 1, sets CDR rates *E_CDR_* in (GtCO_2_/yr) equal to the CO_2_-equivalent of additional agricultural emissions of species *i* at every point in time, using a GWP100 value for methane of 28 [3]. The additional agricultural emissions are calculated from the difference in emissions between the SSP1-2.6 and the alternative scenarios ${(E}_{scenario,i_{agri}}-E_{SSP1-2.6,i_{agri}})$ with *i* being either methane or nitrous oxide emissions, in GtCH_4_/yr or GtN_2_O/yr):

$$E_{CDR}=-\sum GWP100_{i}*{(E}_{scenario,i_{agri}}-E_{SSP1-2.6,i_{agri}})$$ (1)

As the use of GWP100 for short-lived greenhouse gases has limitations [47–51], we explore the use of two additional alternative metrics: the GWP20 and the GWP*. We do not explore the use of GTP100 since the metric leads to larger error, when substituting methane with CO_2_ emissions, than the GWP100 [56]. Since the use of the GWP to calculate equivalent CO_2_ emissions reductions is accurate only for short time horizons [48, 53], we test the effects of using the GWP metric over a time frame of 20 years (GWP20). GWP* denotes an alternative usage of the GWP relating cumulative CO_2_ emissions to date with a change in the rate of emissions of short-lived climate pollutants [55]. We calculate GWP20-based CDR rates as per Eq 1, using GWP20 instead of GWP100. Differently, we calculate GWP*-based CDR as per Eq 2 [55].

$$E_{CDR}=-\frac{\Delta {(E}_{scenario,{CH4}_{agri}}-E_{SSP1-2.6,{CH4}_{agri}})}{\Delta t}\times GWP_{100}\times 100$$ (2)

In a second offsetting approach, we abandon conversion metrics altogether, and instead calculate CDR rates through explicit modelling of effective radiative forcing (we call this the “ERF approach”). In the ERF approach, we explicitly compute CDR requirements using the Finite Amplitude Impulse Response (FaIR) model [64, 65], an open-source reduced complexity carbon-cycle, atmospheric composition and climate model [66]. Starting from input climate-forcing species concentrations or emissions, FaIR calculates the corresponding ERF and temperatures. We numerically invert FaIR to calculate the CDR rates needed to maintain the total ERF on the RCP2.6 pathway even with additional methane and nitrous oxide agricultural emissions, fully accounting for interactions between the different species.

To compare the climate impacts of the two offsetting approaches (GWP100- and ERF-based approach) and to verify whether they meet the RCP2.6 climate requirements, we subtract the CDR rates from the RCP2.6’s fossil fuel CO_2_ emissions. This has the effect to first reduce net positive CO_2_ emissions and then enhance net negative CO_2_. We then calculate, in FaIR, ERF and temperature anomaly. To account for uncertainty in the projections, we compute a 1000-member ensemble simulation using randomized parameters of transient climate response, equilibrium climate sensitivity, pre-industrial sensitivity of carbon sinks, sensitivity to cumulative CO_2_ emissions, sensitivity to temperature change, and ocean temperature response. While the first two are generated from a lognormal distribution informed by the CMIP5 ensemble [65], the other parameters are randomly perturbed by up to 10% from the best estimates reported in Smith et al. (2017) [65]. We then retain only the ensemble members that predict historical temperatures within observational uncertainty, using the method by Thompson et al. (2015) [67]. To allow direct comparison with the UNFCCC temperature goals, we express warming relative to pre-industrial (mean over 1850–1900).

Lastly, we explore CDR costs by calculating the price *p*_*tax*_ per ton agricultural emission (in $/tCO_*2*_*-*eq) necessary to finance the offsetting of agricultural methane emissions (see Eq 3):

$$p_{tax}=\frac{-E_{CDR}∙c_{CDR}}{\sum GWP100_{i}*E_{scenario,i_{agri}}}$$ (3)

where *E_CDR_* is the policy-induced amount of CDR (in tCO_2_), *c*_*CDR*_ is the cost of CDR (in $/tCO_2_) and $E_{scenario,i_{agri}}$ denotes total agricultural emissions of species *i* (t*i*). To compute aggregate increase in prices of emissions for both agricultural methane and nitrous oxide emissions, we first convert each *E_i,agri_* in tCO_2_-equivalent using GWP100 as in the IPCC AR5 [3]. This enables direct comparison with existing and planned carbon taxes that are expressed per ton CO_2_-equivalent emissions. The aggregate tax expressed per ton CO_2_ equivalent is not exact due to the imperfect equivalence of this conversion over a time frame different than 100 years. However, the use of the GWP100 conversion metric does not impact the tax itself which is levied on the methane and nitrous oxide emissions and therefore does not rely on the conversion.

We assume a constant, average cost of CDR by $150/tCO_2_ removed, while evaluating the cost uncertainty by considering a range of possible costs, in each year, between $35-235/tCO_2_ removed. These cost assumptions are based on 2050 CDR cost and potential ranges across the literature, meta-analysed by Fuss et al. (2018) in Table 2 [36]. An array of different negative emission technologies is needed to achieve high rates of carbon removal. Lower-cost CDR techniques (with cost estimates below $120/tCO_2_ by 2050), such as afforestation and reforestation, biochar, and soil carbon sequestration, could contribute by 2050 an aggregate estimated removal potential of 1.4–4 GtC/year. An additional 3–14 GtC/year by 2050 could be contributed by higher-cost CDR techniques: enhanced weathering, bioenergy with carbon capture and storage, and direct air carbon capture and storage [36]. We combine the different ranges of estimates for costs and potential of each CDR technique reported by Fuss et al. (2018) [36] to estimate the average cost of the additional CDR envisaged in our analysis (cf. S1 Table). If all technologies contribute to additional CDR rates, their average cost is $100/tCO_2_eq (with a minimum-maximum scenario interval by around $35-170/tCO_2_eq). Since lower-cost options are likely to be exhausted due to the CDR deployed in the SSP1-2.6 alone, the average cost rises to $150/tCO_2_eq (with a range of around $65-235/tCO_2_eq) in a high-cost CDR only scenario. In our analysis, we use this latter value as an estimate of the long-term cost of additional CDR and explore the uncertainty across the whole range of cost scenarios (from lowest possible CDR cost by $35/tCO_2_eq to highest possible cost by $235/tCO_2_eq). Since it is not possible to reliably predict which technologies will prevail and what their exact cost in each year will be, we use in our analyses a static CDR price. This reflects the rationale [36, 39] that at the beginning higher-cost technologies will have prohibitive costs and limited use, while low-cost options such as afforestation and reforestation will be available at large scales and lower prices. In time, the potential for cheaper options will be exhausted and their price will increase, while technological options will profit from economies of scale and learning effects and their price will decrease as their potential increase [39].

**Table 2:** Methane and nitrous oxide intensities and United Stated and global retail prices for beef, milk, and rice.

	Beef		Milk		Rice	
	CH_4_	N_2_O	CH_4_	N_2_O	CH_4_	N_2_O
GHG intensity (kgCO_2_eq/kg)	20.33	13.44	1.41	0.83	1.05	0.45
Source	Opio et al. (2013) [68]		Opio et al. (2013) [68]		Brodt et al. (2014) [69]	
Method	Global supply-chain life-cycle assessment		Global supply-chain life-cycle assessment		Regional supply-chain life-cycle assessment	
GWP100 used * from IPCC AR4	25*	298*	25*	298*	25*	298*
Retail price U.S.	$12.50/kg beef		$0.85/kg milk		$1.58/kg rice	
Method	Average 2018–2020 all beef products		Average 2018–2020		Average 2017–2019	
Source	USDA, 2020 [70]		USDA, 2020 [70]		U.S. Bureau of Statistics, 2020 [71]	
Worldwide retail price	$4.45/kg beef		$1.61/kg milk		$0.39/kg rice	
Method	Global average 2017–2019 all beef products		Average 2017 price of 14 countries		Global average 2017–2019 four types of rice	
Source	World Bank, 2020 [72]		Export Action Global, 2018 [73]		World Bank, 2020 [72]	

We also consider the cost of CDR per unit of three agricultural products associated with methane and nitrous oxide emissions, multiplying the tax price per ton CO_2_-equivalent emission by the aggregate methane and nitrous oxide emission intensity of the agricultural products. This temporarily reintroduces GWP100 in the analysis, which incompletely accounts for equivalencies between emissions. Yet, real taxes are levied on products of which their disaggregated emissions are known. Thus, the use of GWP100 here is undone when calculating the tax per kilogram agricultural products. We perform the analysis for beef, milk, and rice, covering three different types of food (meat, dairy, and grains) closely linked to activities causing the largest share of greenhouse gases within agriculture (enteric fermentation, manure left on pasture, rice cultivation [12, 68]). We use the reported greenhouse gas intensities from Table 2 (expressed in CO_2_ equivalent per kg product) and first update their value in kg per kg CO_2_-equivalent to reflect the IPCC AR4 GWP100 used in this study. In a second step, we multiply the greenhouse gas intensity (in kg CO_2_-eq/kg product) with the proposed tax (in $/kg CO_2_-eq), yielding a price increase (in $/kg product). Considering the entire data processing pipeline, our approach thus undoes the usage of GWP100. Finally, we calculate the tax-induced mean increase in price relative to two different agricultural price scenarios, using the average retail prices globally (over 2017–2019 for beef and rice, and in 2017 for milk) and in the United States (over 2018–2020 for beef and milk, and 2017–2019 for rice) reported in Table 2. We exclusively consider price increases due to the policy, although agricultural commodities prices are expected to increase due to reduced land availability and growing global GDP even in the absence of the policy (in the SSP1-2.6 scenario) and because of climate change (e.g. due to water scarcity) [1, 3, 4, 74]. We do not consider the price elasticity of demand for the different agricultural products, or the potential for on-farm production changes, which ultimately relates these increases in price to reductions in agricultural emissions. A general equilibrium approach would yield insights into the price-driven decrease in emissions, which would affect the amount of CDR required and hence the tax price itself; this, however, introduces substantial additional uncertainties associated with the other future determinants of agricultural prices over the long time period of our study, and is beyond our scope.

## Results

**Fig 2:**
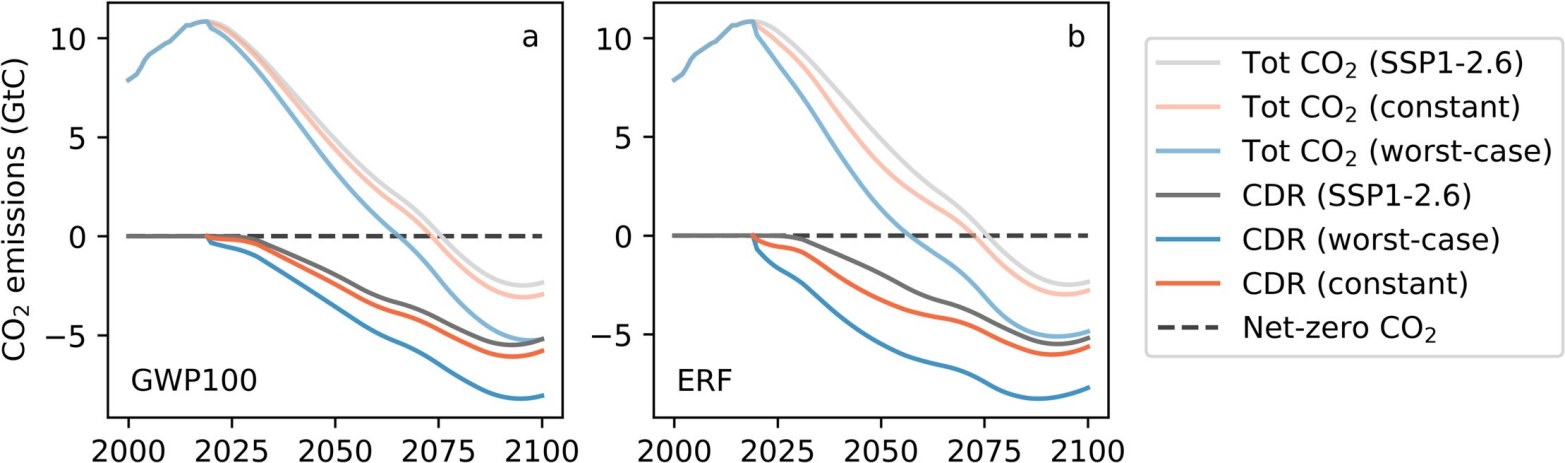
Total CO_2_ emissions and CDR rates under the two offsetting approaches. CO_2_ emissions under the reference scenario (SSP1-2.6) and under a policy scenario in which alternative agricultural emission pathways (constant in orange and worst-case in blue) are offset via CDR. Light coloured lines correspond to the total CO_2_ emissions (including CDR) while darker coloured lines correspond to the total CDR rates. We show the results for two different offsetting approaches: (a) “GWP100” based on the GWP100 metric and (b) “ERF” based on the explicit computation of CDR rates required to achieve the RCP2.6 methane ERF.

Our first results concern the two different approaches to offset additional agricultural methane and nitrous oxide emissions relative to the SSP1-2.6 scenario. Fig 2 portrays the CDR rates and the resulting total CO_2_ emissions required to offset agricultural methane emissions under the two different approaches described above, “GWP100” and “ERF”. The offsetting policies start in year 2020 and envision additional CDR compared to that already present in the RCP2.6 (dark grey line). S1 Fig shows the additional CDR due to the offsetting scheme only.

Both offsetting approaches advance the CDR onset, and have greater annual CDR rates, compared to the SSP1-2.6 scenario. In SSP1-2.6, CDR rates surpass 0.1 GtC/yr in 2030, whereas this occurs already in 2020 for all other scenarios. There are marked differences between the CDR timing and rates under the two offsetting approaches, for each of the emissions scenarios. With the GWP100-based offsetting approach, additional CDR rates reach maximal values of 0.6 GtC/yr in the constant emissions scenario and 2.9 GtC/yr in the worst-case scenario, by the end of the century. Under the ERF approach, additional CDR rates peak higher and sooner, increasing the total CDR use by almost a factor of two compared to the GWP100-based approach. They reach their maximum values at mid-century, at 1.3 GtC/yr for the constant emissions and 3.5 GtC/yr for the worst-case scenario, and then decline slightly in the second half of the century.

**Fig 3:**
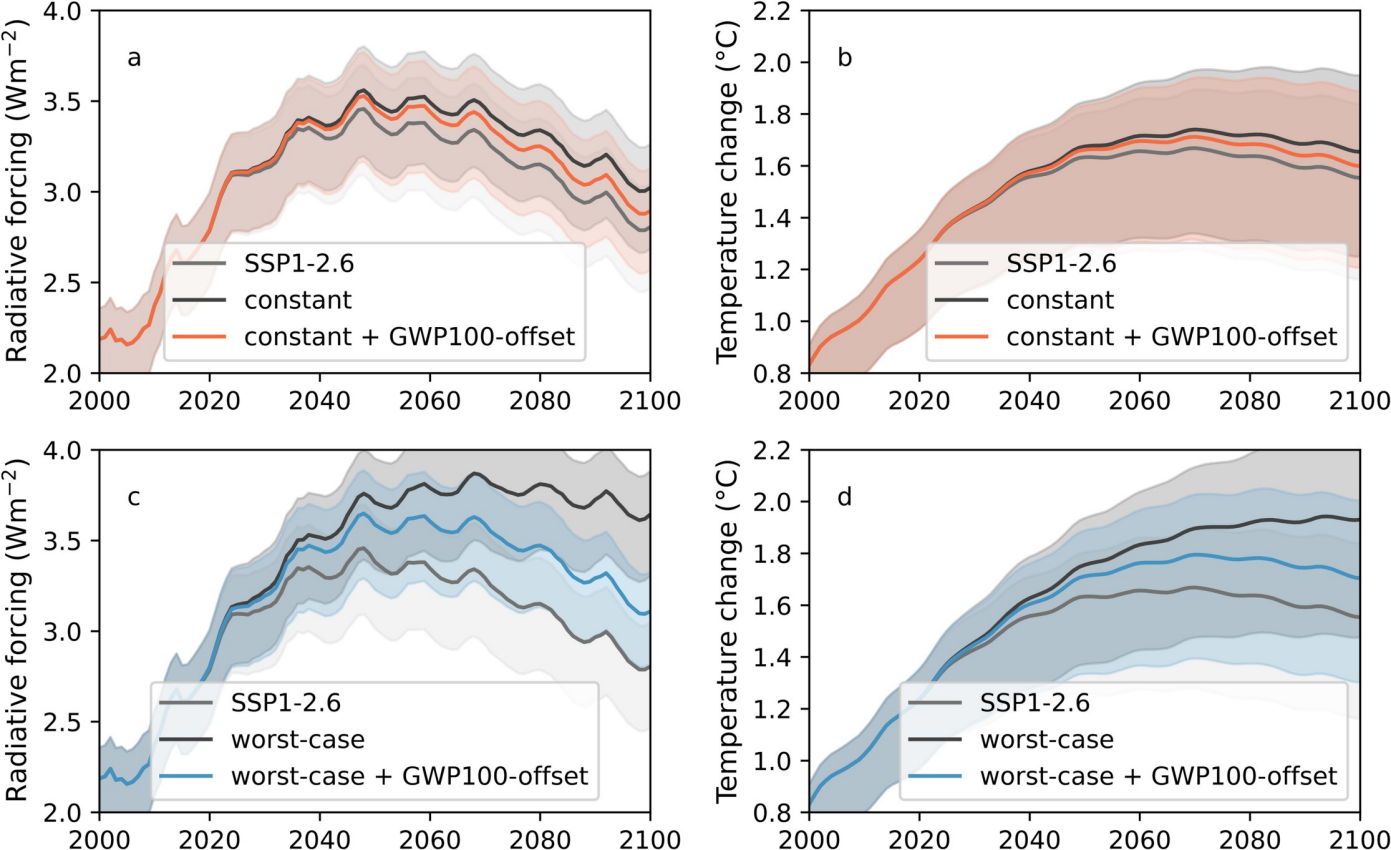
Radiative forcing and temperature anomaly under GWP100 offsetting compared to reference scenarios. Effective radiative forcing (ERF) and temperature anomaly relative to the 1850–1900 average under the SSP1-2.6 scenario (SSP1-2.6), under the alternative emission scenario (constant or worst-case scenario), and under the offsetting scheme using the GWP100 metric. ERF and temperature anomalies result from all forcing agents taken into account by the FaIR model, including natural forcing from solar variability that causes the decadal scale cycle in ERF and temperature anomaly [64, 65]. Thick lines represent simulations with best-estimate parameters [65] whereas shaded areas encompass the 95%-interval of the ensemble simulations. a) ERF under the constant agricultural emission scenario. b) Change in temperature under the constant agricultural emission scenario. c) ERF under the worst-case agricultural emission scenario. d) Change in temperature under the worst-case agricultural emission scenario.

The ERF offsetting approach, by definition, follows RCP2.6 ERF levels and temperature changes in every year (S2 Fig). It avoids additional warming by up to 0.38°C under the worst-case and 0.10°C under the constant agricultural scenarios. By contrast, the GWP100 offsetting approach fails to fully compensate for the additional methane forcing. As shown in Fig 3, around half of the additional temperature rise (0.15°C under the worst-case and 0.05°C under the constant scenarios) remains in 2100 after offsetting according to the GWP100-based approach. While this deviation is smaller than the 95% spread of the ensemble simulations, it points to a systematic under-estimation of offsetting rates determined via GWP100 during the 21^st^ century. When analysing the individual contributions of GWP100-based offsetting, it shows that deviations are almost exclusively due to methane offsetting with negligible contributions from nitrous oxide (cf. S3 and S4 Figs). The use of alternative metrics, such as the GWP20 and the GWP*, only partially resolves these issues (cf. S4 Fig). Both metrics lead to ERF and temperature anomalies below the SSP1-2.6 baseline, thus overestimating the offsetting requirements of additional methane, although these are significantly smaller using GWP*. While performing well in the first 20–40 years after the introduction of the policy but lead, by the end of the century GWP20-based offsetting results in up to 0.2°C lower temperatures than in the SSP1-2.6 scenario. GWP* shows the opposite trend, with larger deviations immediately after the introduction of the policy (up to 0.05°C), and almost no deviation by the end of the century. To conclude, GWP* performs significantly better than the traditional GWPs, yet it leads to slightly more offsetting than necessary. We therefore use the ERF-based offsetting rates in the remainder of this study.

**Fig 4:**
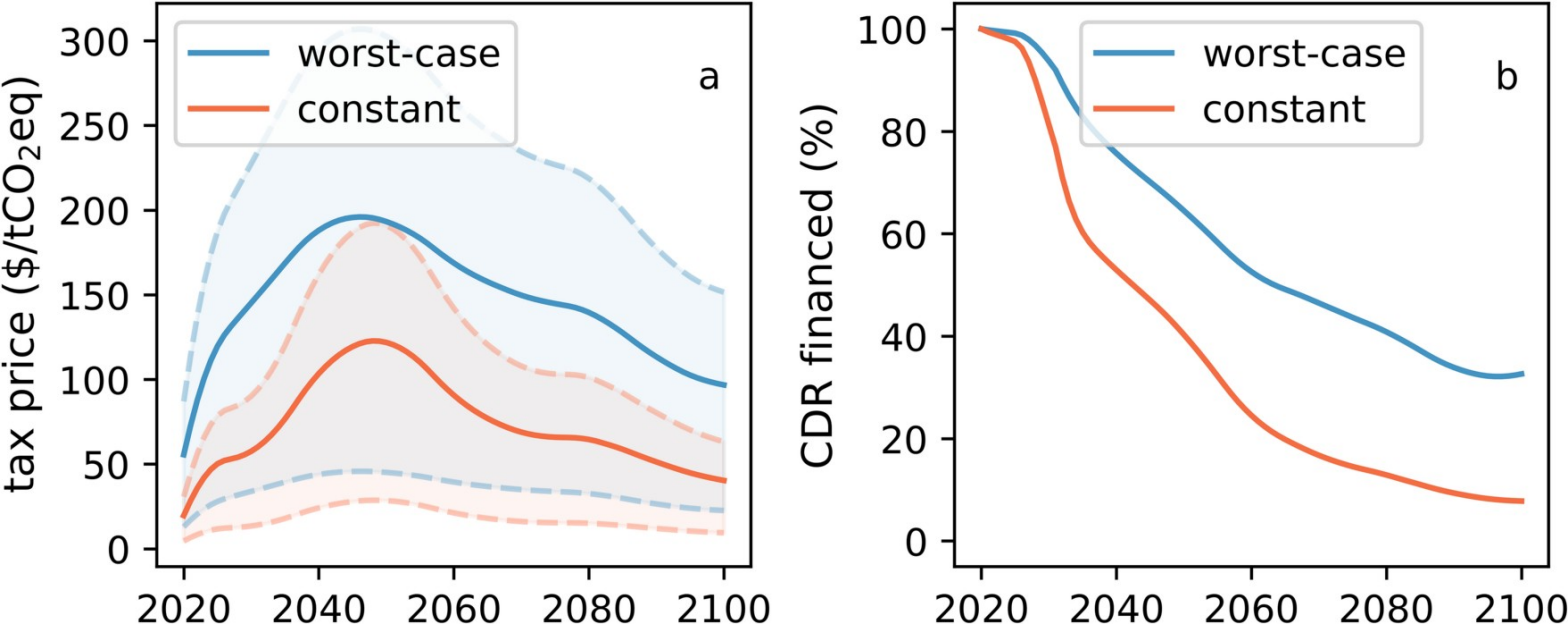
Tax price and percentage of total CDR financed by it. a) Time development of agricultural non-CO_2_ emissions tax price under the constant emissions scenario (orange) and the worst-case emissions scenario (blue). Solid lines are tax prices for a best estimate of future CDR cost by $150/tCO_2_ removed whereas the dotted lines encompass a possible range of CDR costs between $35-235/tCO_2_. b) Percentage of the total CDR (i.e., SSP1-2.6 CDR plus extra CDR to offset additional agricultural emissions) financed via the tax on agricultural emissions under the constant (orange) and worst-case (blue) emission scenario.

Our second set of results concerns the internalization of additional CDR costs via a tax on agricultural methane and nitrous oxide emissions, corresponding to the increase in mitigation cost under the alternative scenarios. Here, we use the costs to offset agricultural emissions via CDR as an estimate of this increase in mitigation cost. Fig 4A shows the temporal evolution of the tax price, commencing with the start of the policy in 2020, under the constant and worst-case emission scenarios. Tax prices substantially vary depending on the CDR cost assumptions, as illustrated by the range encompassed between the dotted lines (representing the range of CDR cost between $35-235/tCO_2_ removed). They also vary in time. After an initial rapid increase up to maximal levels of $123/tCO_2_eq, (constant emissions) and $196/tCO_2_eq, (worst-case) by mid-century, they decline by roughly one half in 2100. This non-linear evolution is mainly driven by the interplay between the difference in agricultural emissions between the SSP1-2.6 and the alternative scenarios and the CDR rates (cf. S1 Fig). In the constant emission scenario, the temporal evolution of CDR rates and tax price are identical by definition. In the “worst-case” scenario, CDR rates grow faster than agricultural emissions in the first half of the 21^st^ century, resulting to increasing tax prices. Towards the end of the 21^st^ century, emissions grow at a slower pace while CDR rates slowly decrease, leading to a decline in tax prices. In absolute terms, however, the “worst-case” tax remains substantially larger than the constant tax at all times.

While the proposed tax would finance all additional CDR to offset agricultural emissions, its relative importance in financing total CDR declines with time. Due to the earlier deployment of CDR compared to SSP1-2.6, nearly all initial CDR is associated with offsetting additional methane and nitrous oxide, and hence a tax on agricultural products would finance nearly all the initial CDR efforts (Fig 4B). As CDR rates increase in the SSP1-2.6 scenario, though, the percent increases over total baseline CDR, and hence the share of CDR financed by such a tax, fall to only 29% (worst-case) and 8% (constant) at the end of the century.

**Fig 5:**
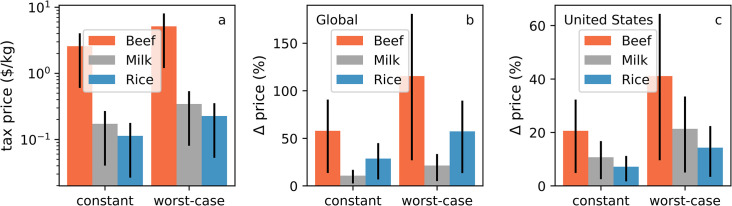
Tax-driven increase in price of agricultural commodities. a) Mean absolute price increase of agricultural products (beef, milk, and rice) due to the introduction of a tax on non-CO_2_ agricultural emissions. b) Relative price increase compared to their current average retail price globally. c) Relative price increase compared to their current average retail price in the United States. Error bars denote the price uncertainty stemming from CDR cost uncertainty in the range of $35-235/tCO_2_ removed.

The introduction of the tax would lead to price increases of agricultural products and can be expressed per kg product. The tax price is influenced by the greenhouse gas intensity of the products, with the highest prices for emissions-heavier products. Fig 5 portrays the effect of a tax on beef, milk, and rice. For beef, the tax would lead to a mean increase in price relative to the retail price that beef had on average in the United States between 2018–2020 –over the 80 years of the policy deployment–by $2.6/kg (constant) and $5.1/kg (worst-case). This corresponds to a mean price increase of 21–41% (Fig 5B). Because the average global price of beef between 2017–2019 was three times lower than the United States one, if the tax were levied homogeneously across the world the global beef price would increase by 58–115% (Fig 5C). Rice prices increase by $0.1–0.2/kg on average, equivalent to 7–14% of its average 2017–2019 retail price in the United States. Globally, the increase in price would be larger (29–57%), since the United States rice price is larger than the global average. Milk shows an average increase by $0.2–0.3/kg (20–40% of their 2018–2020 average retail price in the United States). This increase is smaller (11–21%) globally due to an average higher global milk price.

## Discussion and conclusions

We examine a framework to avoid overshoots of the Paris Agreement’s climate targets in the case of agricultural non-CO_2_ emissions failing to decline. By increasing the rates of CDR compared to those envisioned in the RCP2.6, humanity could compensate for unabated agricultural emissions avoiding up to 0.4°C additional warming. Under alternative agricultural emissions pathways, holding to RCP2.6 total radiative forcing requires up to about twice the CDR already contained in the RCP2.6, corresponding to up to 3.5 GtC/yr additional CDR by mid-century. If the cost of such offsetting were homogeneously levied on global agricultural emissions, on average in the United States it would cause up to 14–41% higher retail price of the agricultural commodities we studied. In a global scenario, average retail prices maximally increase by 57% for rice and 115% for beef.

Our analysis highlights the shortcomings of the approach, common within bodies such as the UNFCCC, of using the GWP100 conversion metric for offsetting purposes. We find that using the GWP100 metric over the 21^st^ century underestimates the short-term climate effects of continuous methane emissions and thus the total CDR requirements by almost 50% and leads to higher ERF and temperature changes than under the RCP2.6 benchmark. This is due to the well-known ambiguity in accounting for peak-warming when using GWP100 with sustained emissions of short-lived climate pollutants [48, 53, 54, 75]. Our findings are hence in line with previous research criticizing the use of the GWP100 when dealing with continuous methane emissions [35, 48, 52, 53, 56, 57, 75, 76] and showing the relative benefits of GWP* [55, 77, 78]. We found deviations from the target mitigation, using GWP100, in the same range of those found by Denison et al. (2019) [52]. The GWP* metric, suggested to better account for the warming effects of cumulative emissions [35, 55], performs significantly better than the GWPs, although it leads to a slight overestimation of CDR requirements. Using the revised formula for GWP* by Cain et al. (2019) [77] may offer a better estimation of the CDR requirements.

We conclude that only an explicit computation of CDR rates derived from the total ERF budget leads to the complete neutralization of the effects of additional agricultural methane. GWP20, GWP100 and GWP* provide estimates of variable quality when benchmarked against explicit modeling. Compared to the RCP2.6 scenario, the ERF-based approach increases the cumulative CDR use by 28% in the constant and 102% in the constant agricultural emission scenarios by 2100. The GWP100 based increases in cumulative CDR are approximately half as large (16% and 63% in the constant and worst-case scenarios, respectively), and thus fail to fully offset the additional climate forcing.

The ERF-based offsetting explored in this study comes, however, with challenges. Firstly, its operationalization within international climate conventions and agreements, such as the UNFCCC and the Paris Agreement, would require a change of current practices relying on the simple heuristic of GWP100-based conversion. Secondly, the initial CDR rates that the approach yields might be too high to be feasible, especially if agricultural emissions follow a worst-case pathway. While currently the maximal estimated capacity for CDR ranges between 0.75–1.5 GtC/yr [79], the ERF approach requires CDR rates over 0.75 GtC/yr already in 2021 and over 1.5 GtC/yr already in 2025 (for the “worst-case” scenario). Such CDR rates are contained in the SSP1-2.6 scenario only starting from 2038–2046, leaving CDR technologies an important time window to develop and scale up. Since the current rates of carbon removal are substantially lower than this maximal estimated capacity, the upscale in CDR in the first years since the introduction of the policy would need to be massive, potentially leading to delays in the early years. On the longer-run, however, the growth rates of CDR converge to those contained in the SSP1-2.6 scenario and, despite additional CDR, higher emission scenarios do not significantly overshoot the maximal CDR capacity in 2100 (5–20 GtC/yr).

Internalizing CDR costs in the form of a tax on agricultural emissions would have two effects: disincentivize the production and consumption of commodities with a negative effect on the climate, hence decreasing agricultural emissions and the amount of CDR needed for their offset, and create an opportunity to finance early deployment of CDR technologies. We examined the latter of these two effects. Average taxes on agricultural non-CO_2_ emissions would range between $59-119/tCO_2_eq, which is in the range of the carbon prices, reported in the IPCC SR15 database [19], needed to limit maximal warming below 2°C. The scheme, which however does not consider the price elasticity of agricultural products, could maximally finance only a small share of the total CDR in 2100 but it would greatly contribute to the financing of CDR’s early deployment. Ensuring early finance-flows to CDR would create an environment of investing security that would lead to more efforts in R&D, technology advances, and learning effects reflecting in sinking technology costs [39]. Yet, there is a large gap between the modelled assumptions of a globally harmonized price on agricultural emissions and the reality of agricultural climate policies. To date, no single country currently imposes carbon prices on agricultural emissions [15]. Public opinion research, as well as the unresolved decades-long debates in New Zealand, the only country discussing their introduction, suggest that stringent agricultural climate policies will encounter large socio-political resistance [15, 29].

Even if the opposition to agricultural emissions prices were overcome and CDR techniques were to grow fast in the next few years, offsetting additional agricultural emissions comes with costs and threats to society. The increased CDR rates have an average direct financial cost corresponding to 0.1–0.2% of the global GDP (as projected in the SSP1-2.6) in 2020, ramping up to 0.2–0.5% by 2050 [19]. This cost is likely to be higher if we considered indirect effects on employment and innovation. Internalizing the cost of the offsetting strategy leads to higher prices of agricultural commodities; rice, for example, is an essential staple food providing over 20% of the calories consumed worldwide [80]. Overly relying on CDR to offset agricultural emissions would thus hit poor households who spend large shares of their incomes on food the hardest [81]. Unless accompanied by other policies to protect the poor, it could lead to an increase in poverty due to agricultural commodities price shocks [82], exacerbating the inherent inequalities in the distribution of impacts due to climate change [83–85]. The approach could moreover entail trade-offs in land-use between low-mitigation agricultural emission scenarios and increased CDR requirements, although land requirements vastly vary among CDR technologies [7, 21, 36, 86].

This paper explored one single possibility, focused on the agricultural sector, to ensure that deviations from the SSP1-2.6 agricultural emissions pathway do not result in additional climate change and to deliver finances to additional CDR rates. We showed the importance of physically sound approaches to correctly offset non-CO_2_ agricultural emissions, revealing the shortcomings of currently used conversion metrics. While the required CDR rates to account for deviations from stringent mitigation pathways directly follow from physical laws, distribution of their economic burden is up to negotiation. The burden does not necessarily need to fall on the agricultural sector, given the potential implication for poverty and food security. A fair distribution should rather also account for historical responsibility as well as current socio-political and economic capacity. To reduce this burden, as well as the risk of relying on unfeasible or unsustainable rates of CDR, strengthening efforts to mitigate agricultural emissions within the limits to food security should remain a climate policy priority.

## Supporting information

S1 Text Supporting discussion. (DOCX)

S1 Fig Additional CDR rates needed to offset agricultural methane and nitrous oxide under the GWP100-based approach (left) and the ERF-based approach (right).
(TIF)

S2 Fig Radiative forcing and temperature anomaly under ERF-based offsetting compared to reference scenarios. Effective radiative forcing (ERF) and temperature anomaly relative to the 1850–1900 average under the SSP1-2.6 scenario, under the alternative emission scenario (constant or worst-case), and under the offsetting scheme using the ERF-based approach metric. Thick lines represent simulations with best-estimate parameters [65] whereas shaded areas encompass the 95%-interval of the ensemble simulations. a) ERF under the constant agricultural emissions scenario. b) Change in temperature under the constant agricultural emissions scenario. c) ERF under the worst-case agricultural emissions scenario. d) Change in temperature under the worst-case agricultural emissions scenario.
(TIF)

S3 Fig Radiative forcing and temperature anomaly under GWP100 offsetting compared to reference scenarios, additional nitrous oxide emissions only. Effective radiative forcing (ERF) and temperature anomaly relative to the 1850–1900 average under the SSP1-2.6 scenario, under the alternative nitrous oxide emission scenario (constant or worst-case), and under the offsetting scheme using the GWP100-based approach metric. Thick lines represent simulations with best-estimate parameters [65] whereas shaded areas encompass the 95%-interval of the ensemble simulations. a) ERF under the constant agricultural nitrous oxide emissions scenario. b) Change in temperature under the constant agricultural nitrous oxide emissions scenario. c) ERF under the worst-case agricultural nitrous oxide emissions scenario. d) Change in temperature under the worst-case agricultural nitrous oxide emissions scenario.
(TIF)

S4 Fig Difference between “target” radiative forcing and temperature anomaly under the use of different conversion metrics to offset methane emissions only. Difference in effective radiative forcing (ERF) and temperature anomalies between the “target” RCP2.6 scenario and scenarios with additional methane emissions under the use of three different conversion metrics: the GWP100, GWP20, and GWP*. Thick lines represent simulations with best-estimate parameters [65] whereas shaded areas encompass the 95%-interval of the ensemble simulations. The dashed line represents the “target” deviation under a perfect offsetting. a) Deviation ERF under the constant agricultural emission scenario. b) Change in temperature under the constant agricultural emission scenario. c) ERF under the SSP3-7.0 agricultural emission scenario. d) Change in temperature under the SSP3-7.0 agricultural emission scenario.
(TIF)

S1 Table CDR cost scenarios by 2050. Different cost scenarios resulting from the literature review by Fuss et al. (2018) [36].
(DOCX)
