# Correction: Offsetting unabated agricultural emissions with CO_2_ removal to achieve ambitious climate targets

**DOI:** 10.1371/journal.pone.0259548

**Published:** 2021-10-28

**Authors:** Nicoletta Brazzola, Jan Wohland, Anthony Patt

In Fig 5, panel c, the grey bars show incorrect values. The authors have provided a corrected version here.

**Fig 5:**
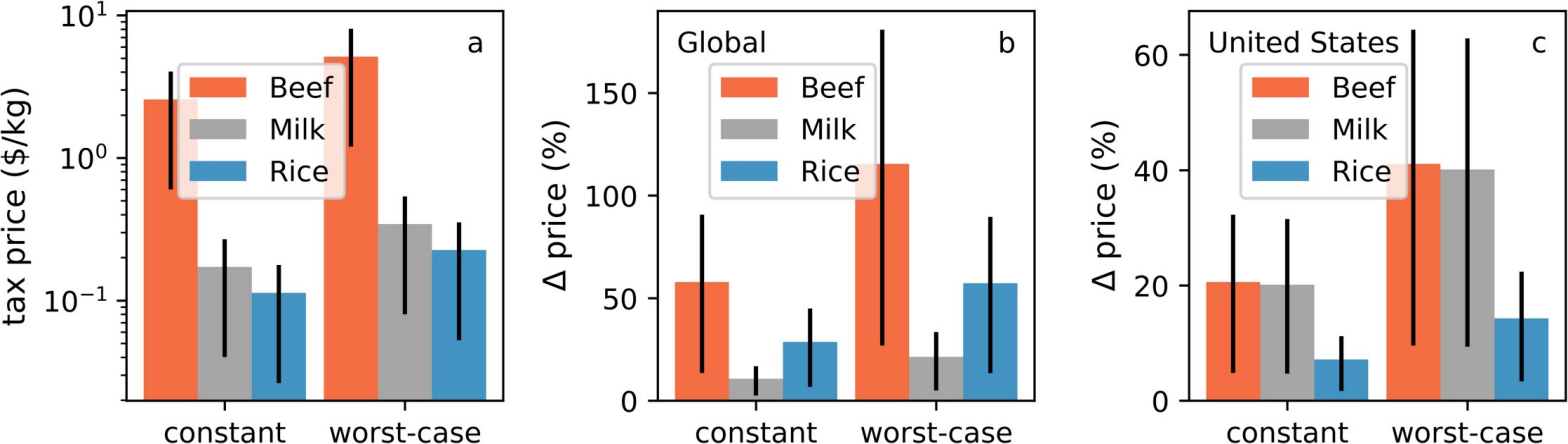
Tax-driven increase in price of agricultural commodities. a) Mean absolute price increase of agricultural products (beef, milk, and rice) due to the introduction of a tax on non-CO2 agricultural emissions. b) Relative price increase compared to their current average retail price globally. c) Relative price increase compared to their current average retail price in the United States. Error bars denote the price uncertainty stemming from CDR cost uncertainty in the range of $35-235/tCO2 removed.

## References

[1] Brazzola N, Wohland J, Patt A (2021) Offsetting unabated agricultural emissions with CO_2_ removal to achieve ambitious climate targets. PLoS ONE 16(3): e0247887. 10.1371/journal.pone.0247887 33730045 PMC7968634

