# Supplementary material for: Offsetting unabated agricultural emissions with CO2 removal to achieve ambitious climate targets
Source: PLoS One. 2021 Mar 17;16(3):e0247887. doi: 10.1371/journal.pone.0247887 (PMC7968634; doi:10.1371/journal.pone.0247887)
Supplement: S1 Text — (DOCX) [file pone.0247887.s001.docx]

**Supplementary Discussion**

Nicoletta Brazzola*^1^, Jan Wohland^1^, & Anthony Patt^1^

Department of Environmental Systems Science, ETH Zurich (Swiss Federal Institute of Technology), Universitätstrasse 16, 8092 Zurich, Switzerland

*corresponding author: nicoletta.brazzola@usys.ethz.ch

CDR rates are different both in trend and magnitude for the GWP100-based approach and for the ERF approach, as shown in Fig 1. With the GWP100-based approach, CDR rates linearly follow the difference in agricultural emissions between the SSP1-2.6 and the alternative emission scenarios. With the ERF approach, CDR rates are designed to force the total ERF on the same pathway as under the SSP1-2.6, accounting for interactions between different species. Acknowledging this complexity gives rise to a generally non-linear relation between CDR and additional agricultural emissions.

The proposed policy begins in 2020 while the alternative agricultural methane and nitrous oxide emission pathways start diverging in 2015. We account for the small difference by evenly distributing the 2015-2020 CDR requirements to the 2020-2100 period. This approach is justified because the 2015-2020 CDR requirements are very small compared to 2020-2100 CDR totals.

**Fig S1. Additional CDR rates needed to offset agricultural methane and nitrous oxide under the GWP100-based approach (left) and the ERF-based approach (right).** Left: GWP100-based offsetting; Right: ERF-based offsetting.

The computation of ERF and temperature under the RCP2.6 and under the ERF offsetting approach for alternative agricultural emission pathways shows small divergences (Fig 2). These add up to a mean difference in ERF by 0.015 Wm^-2^ under the worst-case scenario and by 0.004 Wm^-2^ for the constant scenario, corresponding to a divergence in temperature anomaly by 0.007°C for the worst-case and 0.002°C for the constant scenarios. These differences are two orders of magnitude smaller than the model uncertainty represented by the ensemble simulations due to uncertainties in the parametrization and can therefore safely be neglected. They arise since the policy starts in 2020, five years after agricultural methane and nitrous oxide emissions started deviating from those prescribed by the SSP1-2.6 scenario. They may also be affected by numerical inaccuracies.

**Fig S2.** **Radiative forcing and temperature anomaly under ERF-based offsetting compared to reference scenarios.** ERF and temperature anomaly relative to the 1850-1900 average under the SSP1-2.6 scenario (SSP1-2.6), under the alternative emission scenario (constant and worst-case scenarios), and under the offsetting scheme using the ERF-based offsetting approach. Thick lines represent simulations with best-estimate parameters (Smith et al., 2018) whereas shaded areas encompass the 95%-interval of the ensemble simulations. a) ERF under the constant agricultural emissions scenario. b) Change in temperature under the constant agricultural emissions scenario. c) ERF under the worst-case agricultural emissions scenario. d) Change in temperature under the worst-case agricultural emissions scenario.
