# Supplementary figures and images for: Offsetting unabated agricultural emissions with CO2 removal to achieve ambitious climate targets
Source: PLoS One. 2021 Mar 17;16(3):e0247887. doi: 10.1371/journal.pone.0247887 (PMC7968634; doi:10.1371/journal.pone.0247887)

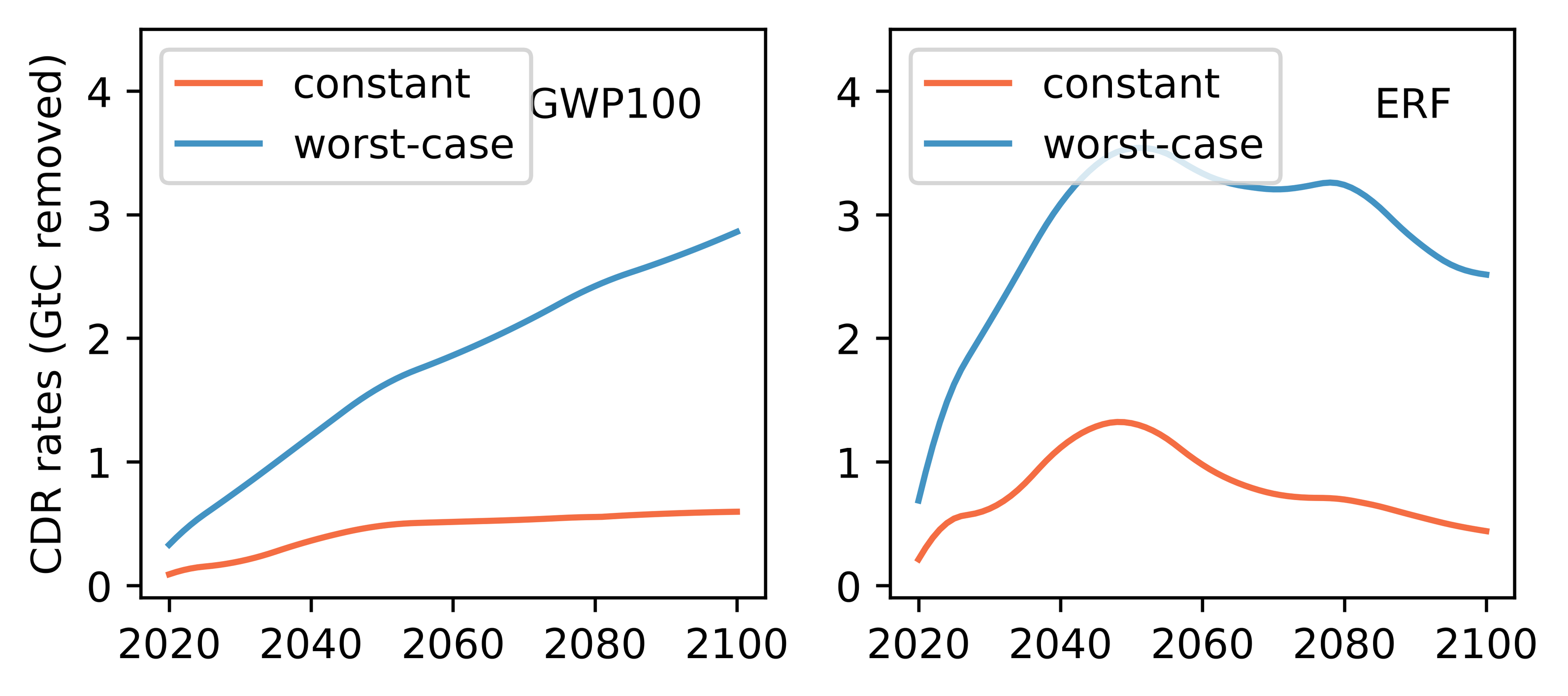

Supplement: S1 Fig — Additional CDR rates needed to offset agricultural methane and nitrous oxide under the GWP100-based approach (left) and the ERF-based approach (right). (TIF) [file pone.0247887.s002.tif]

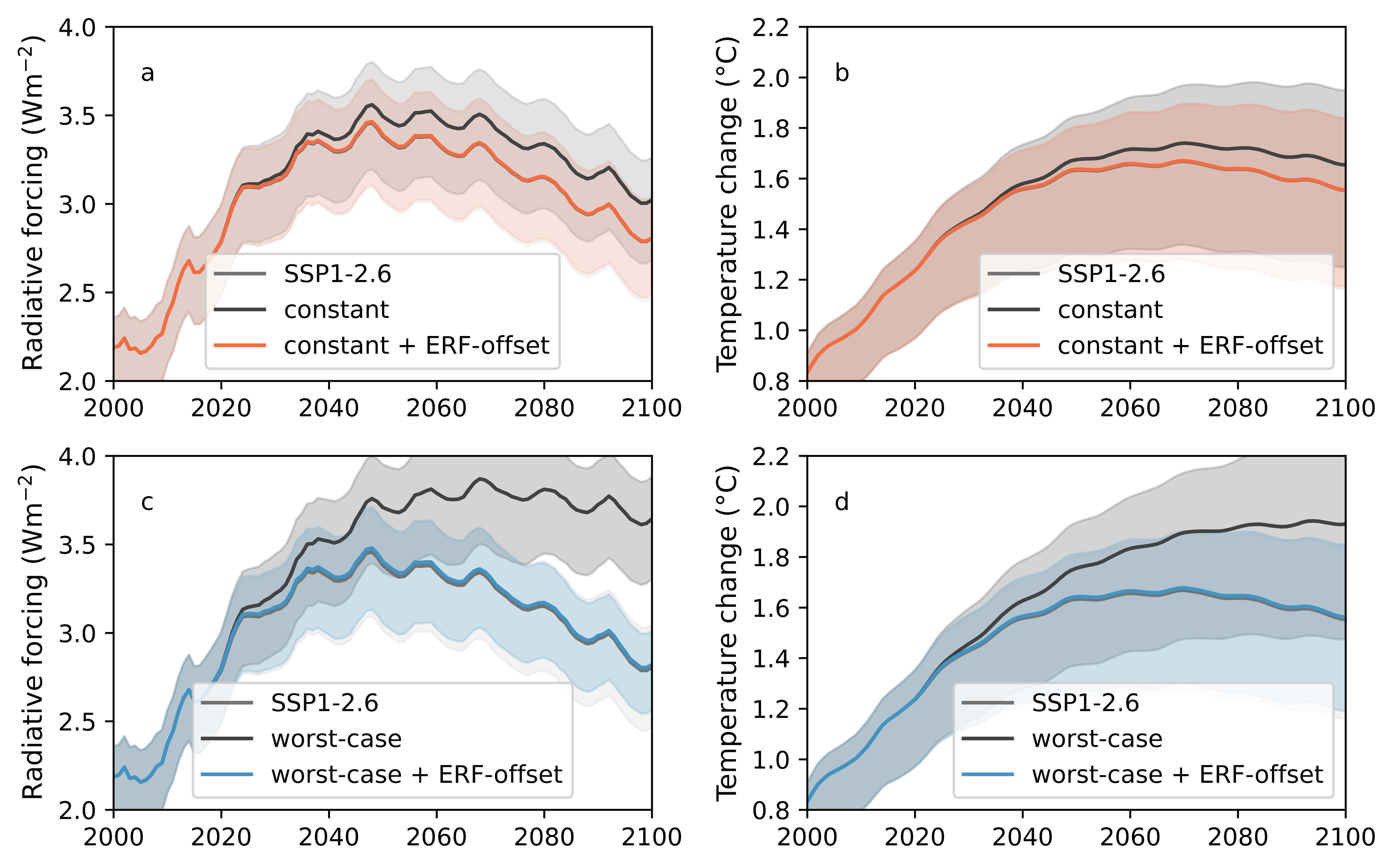

Supplement: S2 Fig — Effective radiative forcing (ERF) and temperature anomaly relative to the 1850–1900 average under the SSP1-2.6 scenario, under the alternative emission scenario (constant or worst-case), and under the offsetting scheme using the ERF-based approach metric. Thick lines represent simulations with best-estimate parameters [65] whereas shaded areas encompass the 95%-interval of the ensemble simulations. a) ERF under the constant agricultural emissions scenario. b) Change in temperature under the constant agricultural emissions scenario. c) ERF under the worst-case agricultural emissions scenario. d) Change in temperature under the worst-case agricultural emissions scenario. (TIF) [file pone.0247887.s003.tif]

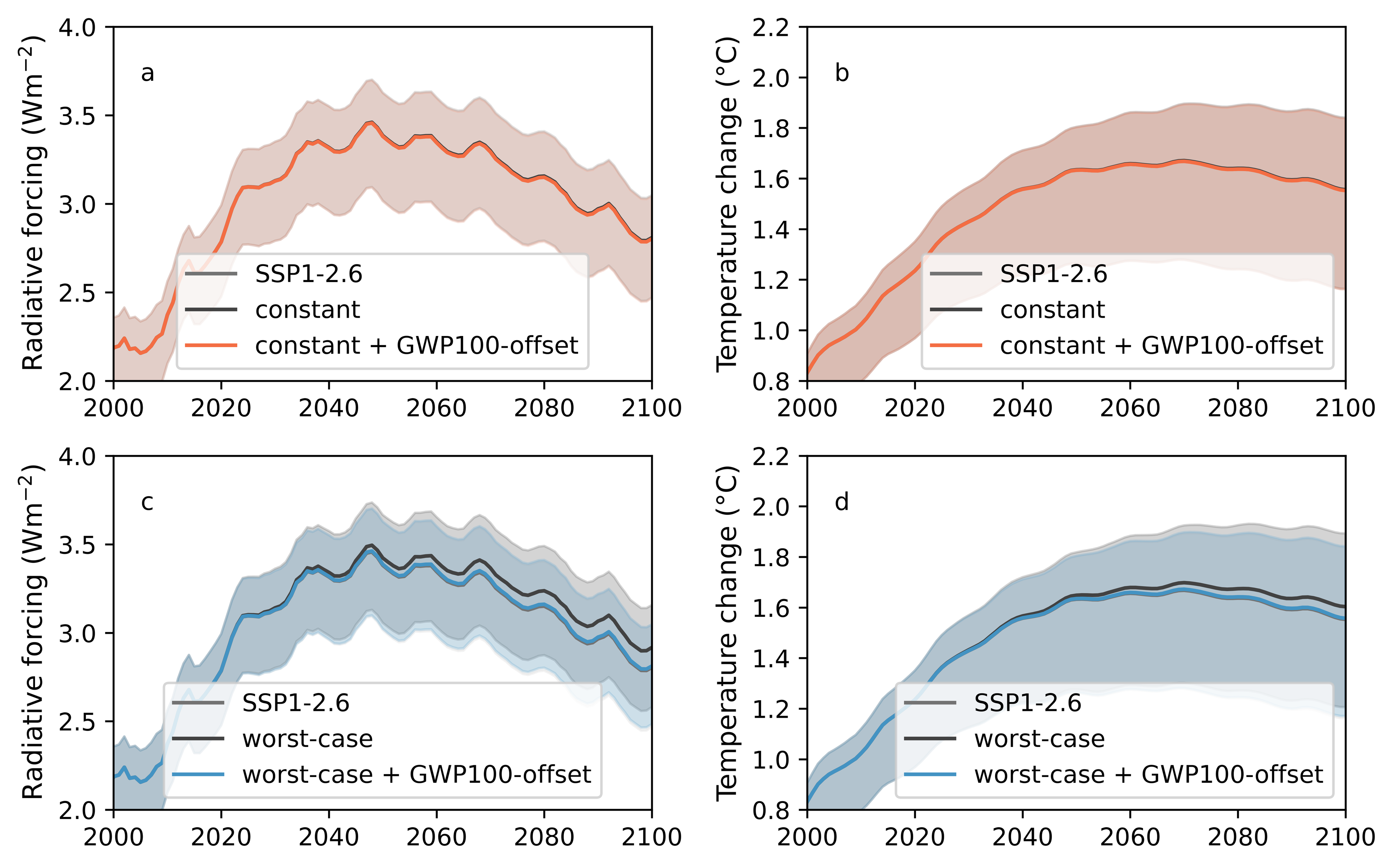

Supplement: S3 Fig — Effective radiative forcing (ERF) and temperature anomaly relative to the 1850–1900 average under the SSP1-2.6 scenario, under the alternative nitrous oxide emission scenario (constant or worst-case), and under the offsetting scheme using the GWP100-based approach metric. Thick lines represent simulations with best-estimate parameters [65] whereas shaded areas encompass the 95%-interval of the ensemble simulations. a) ERF under the constant agricultural nitrous oxide emissions scenario. b) Change in temperature under the constant agricultural nitrous oxide emissions scenario. c) ERF under the worst-case agricultural nitrous oxide emissions scenario. d) Change in temperature under the worst-case agricultural nitrous oxide emissions scenario. (TIF) [file pone.0247887.s004.tif]

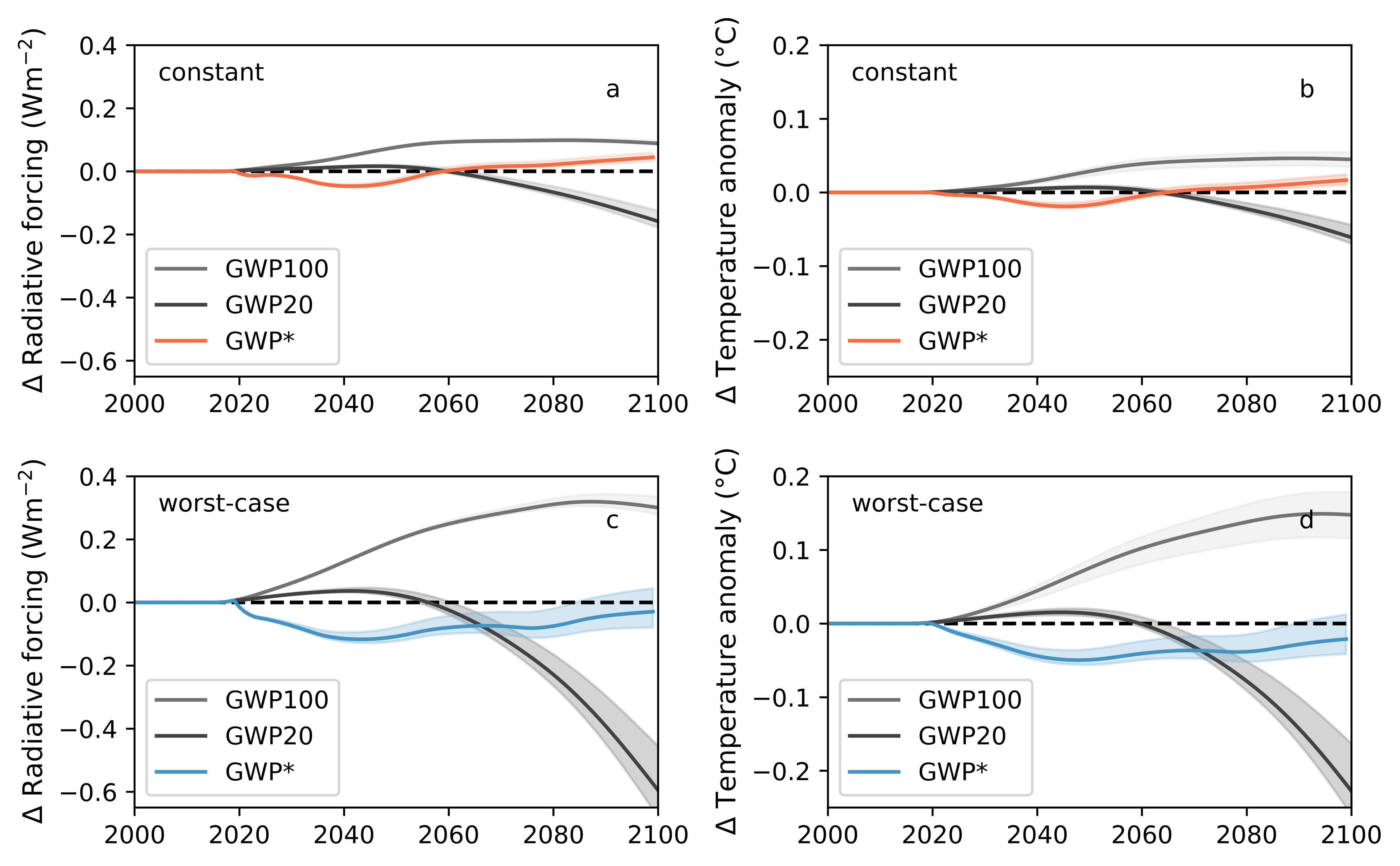

Supplement: S4 Fig — Difference in effective radiative forcing (ERF) and temperature anomalies between the “target” RCP2.6 scenario and scenarios with additional methane emissions under the use of three different conversion metrics: the GWP100, GWP20, and GWP*. Thick lines represent simulations with best-estimate parameters [65] whereas shaded areas encompass the 95%-interval of the ensemble simulations. The dashed line represents the “target” deviation under a perfect offsetting. a) Deviation ERF under the constant agricultural emission scenario. b) Change in temperature under the constant agricultural emission scenario. c) ERF under the SSP3-7.0 agricultural emission scenario. d) Change in temperature under the SSP3-7.0 agricultural emission scenario. (TIF) [file pone.0247887.s005.tif]
