# Supplementary material for: Offsetting unabated agricultural emissions with CO2 removal to achieve ambitious climate targets
Source: PLoS One. 2021 Mar 17;16(3):e0247887. doi: 10.1371/journal.pone.0247887 (PMC7968634; doi:10.1371/journal.pone.0247887)
Supplement: S1 Table — Different cost scenarios resulting from the literature review by Fuss et al. (2018) [36]. (DOCX) [file pone.0247887.s006.docx]

**S1 Table.** Different cost scenarios resulting from the literature review by Fuss et al. (2018) [36]

|  | Minimal cost | | Maximal cost | |
| --- | --- | --- | --- | --- |
|  | All types of CDR | Higher-cost CDR only | All types of CDR | Higher-cost CDR only |
| Minimal potential | $36.25/tCO_2_eq | $66.67/tCO_2_eq | $155.83/tCO_2_eq | $216.67/tCO_2_eq |
| Maximal  potential | $51.95/tCO_2_eq | $85.71/tCO_2_eq | $171.54/tCO_2_eq | $235.71/tCO_2_eq |
| Average of all scenarios | All types of CDR  $103.89/tCO_2_eq | | Higher-cost CDR only  $151.19/tCO_2_eq | |
